# Supplementary material for: Validation of automatic monitoring of feeding behaviours in sheep and goats
Source: PLoS One. 2023 May 18;18(5):e0285933. doi: 10.1371/journal.pone.0285933 (PMC10194855; doi:10.1371/journal.pone.0285933)
Supplement: S1 Table — Table 2: Descriptions of true and false positive and negative seconds of feeding and ruminating. Table 3: Model estimates of the agreement parameters and model comparison results for significance of fixed effects. (DOCX) [file pone.0285933.s001.docx]

# S1 Supplementary tables

**Supplementary Table 1| Table of components and the chemical composition per kilogram dry matter (DM) of the three mixed rations: first- and second-cut hay (HH), grass silage and hay (GH), and maize silage and grass silage (MG).**

|  | Unit | HH | | GH | | MG | |
| --- | --- | --- | --- | --- | --- | --- | --- |
| first-cut grass hay | %/ kg DM | 50 | | 50 | | -- | |
| second-cut grass hay | %/kg DM | 50 | | -- | | -- | |
| grass silage | %/ kg DM | -- | | 50 | | 55 | |
| maize silage | %/ kg DM | -- | | -- | | 40 | |
| alfalfa hay | %/ kg DM | -- | | -- | | 5 | |
| *mean (±SD)* |  |  | |  | |  | |
| dry matter | kg/ kg fresh matter | 0.89 | ±0.0 | 0.65 | ±0.0 | 0.52 | ±0.0 |
| organic matter | g/kg DM | 926.2 | ±2.7 | 918.6 | ±3.4 | 933.6 | ±2.6 |
| crude protein | g/ kg DM | 90.27 | ±2.6 | 90.17 | ±2.6 | 92.3 | ±3.4 |
| ADF | g/ kg DM | 277.6 | ±8.53 | 301.1 | ±13.5 | 257.0 | ±12.3 |
| NDF | g/ kg DM | 470.8 | ±12.5 | 484.4 | ±12.5 | 421.0 | ±15.6 |
| NEL* | MJ/ kg DM | 5.5 | ±0.0 | 5.1 | ±0.1 | 5.6 | ±0.1 |
| ADPE* | g/ kg DM | 79.1 | ±0.9 | 68.5 | ±1.0 | 66.2 | ±0.9 |
| ADPN* | g/ kg DM | 56.5 | ±1.7 | 56.5 | ±1.7 | 57.7 | ±2.1 |

*ADF: acid detergent fibre, NDF: neutral detergent fibre, NEL: net energy for lactation, APDE: absorbable protein at the duodenum limited by rumen fermentable energy, APDN: absorbable protein at the duodenum limited by rumen fermentable nitrogen; *calculated according to*

**Supplementary Table 2| Description of true and false positive and negative seconds of feeding and ruminating.**

| Evaluating seconds for feeding behaviour | | | | | |
| --- | --- | --- | --- | --- | --- |
| **Viewer2 classified behaviour** | **observed behaviour** | | | | |
|  | **Feeding** | **Ruminating** | **No oral behaviour** | **other oral behaviour** | **Drinking** |
| **Feeding** | TP | FP | FP | FP | FP |
| **Ruminating** | FN |  |  |  |  |
| **No oral behaviour** | FN |  | TN | TN | TN |
| Evaluating seconds for ruminating behaviour | | | | | |
| **Viewer2 classified behaviour** | **observed behaviour** | | | | |
|  | **Feeding** | **Ruminating** | **No oral behaviour** | **other oral behaviour** | **Drinking** |
| **Feeding** |  | FN |  |  |  |
| **Ruminating** | FP | TP | FP | FP | FP |
| **No oral behaviour** |  | FN | TN | TN | TN |

**Supplementary Table 3| Model estimates of the agreement parameters and model comparison results for significance of fixed effects.**

| **Parameter** | **Factor** | **Level** | **Estimate** | **Std. Error** | **χ^2^** | **p-value** |
| --- | --- | --- | --- | --- | --- | --- |
| formula: | *parameter ~ behaviour + species + (1\|name) + (1\|condition) + (1\|frequency)* | | | | | |
| **Accuracy** | (intercept) | goat feed | **0.88** | ±0.04 |  |  |
|  | species | sheep | 0.02 | ±0.02 | 1.63 | 0.20 |
|  | behaviour | ruminate | -0.03 | ±0.01 | 8.71 | < 0.01 |
| **Sensitivity** | (intercept) | goat feed | **0.89** | ±0.04 |  |  |
|  | species | sheep | 0.06 | ±0.03 | 6.06 | 0.01 |
|  | behaviour | ruminate | -0.20 | ±0.02 | 46.35 | < 0.01 |
| **Specificity** | (intercept) | goat feed | **0.74** | ±0.08 |  |  |
|  | species | sheep | 0.00 | ±0.03 | 0.00 | 0.99 |
|  | behaviour | ruminate | 0.23 | ±0.03 | 47.08 | < 0.001 |
| **Precision** | (intercept) | goat feed | **0.80** | ±0.06 |  |  |
|  | species | sheep | 0.02 | ±0.02 | 1.14 | 0.29 |
|  | behaviour | ruminate | 0.16 | ±0.02 | 34.44 | < 0.01 |
| formula: | *parameter ~ behaviour + condition + (1\|name) + (1\|species)* | | | | | |
| **Accuracy** | (Intercept) | pasture feed | 0.86 | ±0.02 |  |  |
|  | condition | barn | 0.00 | ±0.02 | 2.33 | 0.13 |
|  | behaviour | ruminate | -0.07 | ±0.01 | 12.21 | < 0.001 |
|  | condition:behaviour | barn:ruminate | 0.08 | ±0.02 | 8.22 | < 0.01 |
| **Sensitivity** | (Intercept) | pasture feed | 0.93 | ±0.04 |  |  |
|  | condition | barn | -0.04 | ±0.04 | 4.65 | 0.03 |
|  | behaviour | ruminate | -0.21 | ±0.03 | 47.37 | < 0.001 |
|  | condition:behaviour | barn:ruminate | -0.05 | ±0.06 | 0.85 | 0.36 |
| **Specificity** | (Intercept) | pasture feed | 0.59 | ±0.03 |  |  |
|  | condition | barn | 0.25 | ±0.05 | 13.83 | < 0.001 |
|  | behaviour | ruminate | 0.36 | ±0.04 | 46.90 | < 0.001 |
|  | condition:behaviour | barn:ruminate | -0.22 | ±0.07 | 10.44 | < 0.01 |
| **Precision** | (Intercept) | pasture feed | 0.59 | ±0.03 |  |  |
|  | condition | barn | 0.25 | ±0.05 | 20.78 | < 0.001 |
|  | behaviour | ruminate | 0.36 | ±0.04 | 30.67 | < 0.001 |
|  | condition:behaviour | barn:ruminate | -0.22 | ±0.07 | 20.76 | < 0.001 |
| formula: | *parameter ~ behaviour + frequency + (1\|name) + (1\|species)* | | | | | |
| **Accuracy** | (Intercept) | 10 Hz feed | 0.86 | ±0.02 |  |  |
|  | frequency | 20 Hz | 0.05 | ±0.02 | 15.16 | < 0.001 |
|  | behaviour | ruminate | 0.00 | ±0.02 | 0.38 | 0.54 |
|  | frequency:behaviour | 20 Hz:ruminate | 0.02 | ±0.03 | 0.42 | 0.52 |
| **Sensitivity** | (Intercept) | 10 Hz feed | 0.89 | ±0.05 |  |  |
|  | frequency | 20 Hz | 0.02 | ±0.06 | 3.16 | 0.08 |
|  | behaviour | ruminate | -0.26 | ±0.06 | 17.55 | < 0.001 |
|  | frequency:behaviour | 20 Hz:ruminate | 0.12 | ±0.08 | 2.26 | 0.13 |
| **Specificity** | (Intercept) | 10 Hz feed | 0.85 | ±0.02 |  |  |
|  | frequency | 20 Hz | 0.07 | ±0.02 | 4.75 | 0.03 |
|  | behaviour | ruminate | 0.14 | ±0.02 | 23.21 | < 0.001 |
|  | frequency:behaviour | 20 Hz:ruminate | -0.07 | ±0.03 | 4.38 | 0.04 |
| **Precision** | (Intercept) | 10 Hz feed | 0.67 | ±0.04 |  |  |
|  | frequency | 20 Hz | 0.10 | ±0.06 | 1.20 | 0.27 |
|  | behaviour | ruminate | 0.30 | ±0.06 | 22.94 | < 0.001 |
|  | frequency:behaviour | 20 Hz:ruminate | -0.11 | ±0.09 | 1.88 | 0.17 |
