# Supplementary material for: Validation of automatic monitoring of feeding behaviours in sheep and goats
Source: PLoS One. 2023 May 18;18(5):e0285933. doi: 10.1371/journal.pone.0285933 (PMC10194855; doi:10.1371/journal.pone.0285933)
Supplement: S1 File — (DOCX) [file pone.0285933.s002.docx]

## S2 Supplementary Method description| Analysis of feed samples

Samples of the feed rations were taken daily. Samples were dried at 60°C for 48 hours to calculate the dry matter content as percentage of fresh matter. For the subsequent chemical analyses, dried samples were pooled per experimental group and ground to pass a 1-mm screen (Brabender rotary mill; Brabender GmbH & Co. KG, Duisburg, Germany). Feed samples were analyzed for exact dry mass content by heating at 105˚C for three hours (prepASH, Precisa Gravimetrics AG, Dietikon, Switzerland) and then incinerating at 550°C until a stable mass was reached to determine the ash content according to ISO 5984_2002. Crude fiber content was determined gravimetrically (ISO 6865:2000) by incineration of residual ash after acid and alkaline digestions using a fiber analyzer (Fibretherm Gerhardt FT-12, C. Gerhardt GmbH & Co. KG, Königswinter, Germany).  The Neutral detergent fiber (αNDF; ISO 16472:2006) and acid detergent fiber (ADF; ISO 13906:2008) contents were analyzed with the same fiber analyzer (Fibretherm Gerhardt FT-12) and were expressed without residual ash. Neutral detergent fiber (αNDF) was evaluated with heat stable amylase and sodium sulfite and expressed without residual ash after incineration at 600°C for three hours.
