# Supplementary material for: Validation of automatic monitoring of feeding behaviours in sheep and goats
Source: PLoS One. 2023 May 18;18(5):e0285933. doi: 10.1371/journal.pone.0285933 (PMC10194855; doi:10.1371/journal.pone.0285933)
Supplement: Attachment — Submitted filename: answers to Editor and Reviewers.docx [file pone.0285933.s005.docx]

# Answer to the Editor

Thank you for your feedback and comments. We have revised the manuscript, taking careful account of all the reviewers comments.

According to your comments, we added the copyright information on the figures:

We added the copyright indication on figure 1 and 2 and we added the source to the picture Supplementary Figure 1.

With this information, we have the permission to use Google maps screenshots in journals, as you can read here: <https://about.google/brand-resource-center/products-and-services/geo-guidelines/#google-maps>

# Answer to Reviewer #1

General comments

Answer:
Thank you very much for your valuable feedback and recommending our work for publication. We have revised the manuscript, taking careful account of all your comments.

Some specific comments as line by line below:

Title: Since the manuscript is mainly focusing on validating the JAM-R technology, I think this should be clarified in the manuscript title. Perhaps along the lines of: “Validation of feeding and rumination behaviours using an automatic jaw-movement recording system in sheep and goats.”

(1) We changed the titel to “Validation of automatic monitoring of feeding behaviours in sheep and goats”.
(2) **Abstract:**
    Overall, the abstract reads well. In light of my comments below, lines 36-38 may need to be omitted as they currently are not properly linked to the overarching aim of the paper. I think that the paper is mainly focused on validating the technology more so than describing sheep and goat feeding behavior which as been done previously. The novelty lies in the validation aspect.

Answer:
We changed the focus of the feeding experiment and instead of reporting differences between sheep and goats we report number of technical failures and information on missing data. The main result of this assessment was instead included in the Abstract.

Lines 36-37: “Applying the JAM-R in a feeding experiment, 71 % of the recordings executed were defined as technically error-free and produced plausible values of feeding behaviours.”

**Introduction:**

(3) R1: Ln 79-80: Change to past tense. E.g. ‘The JAM-R technology has previously been validated for dairy cows’.

Answer:
It now reads (lines 69-70): “The JAM-R technology has previously been tested and validated for dairy cows.”

(4) R1: Ln 102-103: Consider rewording ‘checked’ to ‘investigated’. Also, I find this last aim of the paper somewhat strange. A better aim would be to investigate if the JAM-R technology is consistently accurate and precise between commonly used goat and sheep MR’s, and then compared the values to the existing literature regardless. As it currently sits, it’s purely descriptive and a stand-alone-aim not linked to validating the JAM-R technology. If you have additional data for the feed trial, I would highly encourage to analyze this and add it to the paper.

Answer:
The word “checked” was changed to “investigated” in line 90.

We now emphasize on issues of applicability and issues with fitting the halter (see Revisions of 2.5. M&M and 3.3 Results).

It now reads (lines 93-96): “Additionally, the system was applied to sheep and goats in a feeding experiment on three different mixed rations (MRs) to assess the plausibility of obtained values and report frequencies of technical failure and possible problems for the welfare of the animals due to the wearing of the halter.”

**Material and methods:**

(5) Overall, the material and method reads a bit jumbled and could need some clarification and separation between methods, data editing and statistics.

Answer:
The Material and Methods and the results sections were reorganized and information added. Here you see the new table of content of the Material and Methods and the Results section including line numbers.

**2	Material and Methods	7**

2.1	Animals and housing	8

2.1.1	Non-experimental condition	8

2.1.2	Pasture	9

2.1.3	Experimental pens	10

2.1.4	Feeding experiment for application of JAM-R	11

2.2	Behaviour recording by observation	11

2.3	Automatic behaviour recording	13

2.3.1	MSR-jaw-movement recording system (JAM-R)	13

2.3.2	Viewer2 behaviour classification software	15

2.4	Comparison of the behaviour recording methods	16

2.4.1	Data management	16

2.4.2	Parameters of agreement	17

2.4.3	Effects of species, condition and recording frequency on agreement parameters	18

2.5	Application of JAM-R in a feeding experiment	20

2.5.1	Description of the feeding experiment	20

2.5.2	Assessment of welfare issues and data quality	21

2.5.3	Estimation of variance of data loggers	22

**3	Results	22**

3.1	Comparison of the behaviour recording methods	22

3.2	Parameters of agreement	24

3.2.1	Effect of condition: Pasture versus barn (10 Hz)	25

3.2.2	Effect of recording frequency: 10 Hz versus 20 Hz	25

3.3	Application of JAM-R in a feeding experiment	26

3.3.1	Values of feeding and ruminating behaviour	26

3.3.2	Assessment of welfare issues and data quality	26

3.3.3	Variance of data loggers on output variables of the Viewer2	27

(6) R1: Ln 122-125: I would consider moving the breed information up after the first sentence of this section (ln 116).

Answer:
Breed information was moved in the paragraph (Lines 114-115).
Abbreviations for breeds names were introduced to later report the number of animals per breed: “The sheep were of the dairy breeds Lacaune (LC) and East-Friesian (EF). The goats were of the dairy breeds Saanen (SA), Chamois Coloured (CC) and hybrids of these two breeds (HY).”

(7) R1: Ln 147: Redundant line, information given previously.

Answer:
The line was deleted.

(8) R1: Ln 142-158: I am a bit uncertain of the numbers of replicates used. Five or eight? But 1 replicate for animals on pasture? Please clarify.

Answer:
The paragraph was rephrased for better understanding.

It now reads (lines 145-149): “**For the observations of the goats on five consecutive days,** the plot was extended by 10 m x 30 m (total plot = 10 m x 50 m). When the observation period of the goats was finished, the sheep were then moved to this plot, and it was extended by an additional 10 m x 10 m (total plot = 10 m x 60 m; Supplementary Figure 1). On this plot**, the behaviour observation of sheep was conducted for six consecutive days**.”

(9) R1: Ln 168: This is an unfortunate limitation of the study. It would have been great to have several observers and their inter-reliability scores presented to reduce the risk of bias regardless of the experience of the observer, especially as the ethograms differed slightly between species. Was any intra-reliability score conducted?

Answer:
The ethograms for the two species were exactly the same (Table 1, line188). May you please state where you found any contrary information so we can correct this?

We included a paragraph on inter-observer reliability for the video analysis (lines 180-183): “Video behaviour observation was assessed for agreement with another trained observer (R.B.) for 21600 seconds of two videos. R.B. reached an agreement of 97.6 - 98.2% for seconds observed as feeding by A.D. and 98.5 - 99.9% for seconds of rumination of A.D..”

(10) R1: Ln 167-241: This section of the manuscript is a bit hard to follow. Consider making a main header named “Statistical analysis” for only the statistical approach and make an appropriate sub-headers for the accuracy, sensitivity, specificity and precision formulas. Perhaps the sub-header could be simply: ‘Validation formulae”?
     The other information should be restructured and placed in material and methods under a sub-header such as ‘‘Data classification and management” or similar for ease of flow and readability.

Answer:
M&M section was restructured and headers renamed. We hope it is now more easy to follow. Please see point (5) for the table of contents.

(11) Ln 285-299: This section could use some clarification, please spell out the parameters and factors used (or the LME formula) in each significant step to help the reader follow the path of analysis.

Answer:
We added model parameters and formulas in the section (line 305 – 335)

(12) Ln 300: Consider renaming this section as the second project/trial and move this section up before the statistical analysis section.

Answer:
The section was renamed as “2.5 Application of JAM-R in a feeding experiment” and subheadings were introduced with explaining the (new) purpose of this section.

(13) Ln 326: If these MR’s are commonly used, perhaps the full chemical analyses section may be redundant in the scope of the paper and could be omitted. As mentioned earlier, if the JAM-R was tested on different MR’s rather than only providing descriptive data on the different MR’s per species, that would be better aligned with the scope of the paper.

Answer:
The feed analyses and values had been moved to the supplementary (Supp. Table 1 and Supp. Methods Description).

(14) Ln 342 and elsewhere: N= 345,657. Change to a comma for all numbers except for the decimals ‘.’.

Answer:
done.

**Results:**
Rename the headers to reflect the structure in the manuscript after suggested changes above.

Answer:
The headers were renamed. See point (5) for the table of contents.

(15) Ln 356: What statistical analysis was done here? A chi-square? Please clarify in the statistical analysis section. Currently only LME and ANOVA are listed.

Answer:
The R function anova () does not perform an ANOVA.

It is now explained in the methods section (lines 331-334): “The models were stepwise reduced by the interaction and then by each fixed effect and compared with the full model. This was done using the anova function (25), which performs a χ2 test based on the restricted maximum likelihood method to determine whether the fixed effect omitted in the reduced model is statistically significant for the model fit.”

(16) Ln 363: are the (+/-) in SE or SD? Please clarify.

Answer:
It is the standard error. The missing information was added in line 402.

(17) Ln 389-410: This section is presenting data not specifically related to the main aim or objective of the paper. The presentation of the estimated feeding behaviours for each species is somewhat redundant when not compared to manual observation. As it stands, the results presented are bound to be more linked to housing and feed rather than the technology.

Answer:
The focus and reported data of this section was changed in relation to several issues of applicability. It now also reports reasons and frequencies of missing data, any technical errors during the application and potential problems arising with the fit of the halters. The focus of the statistical model is now about magnitude of the random effects, especially the variation explained among loggers. The section header was renamed (line 430) “3.3	Application of JAM-R in a feeding experiment”. The headers of the subsections are now (line 431) “3.3.1 Values of feeding and ruminating behvaiour”, (line 442) “3.3.2 Assessment of welfare issues and data quality” and (line 462) “3.3.3 Variance of data loggers on output variables of the Viewer2”.

**Discussion:**
Overall, I think the discussion reads nicely with the study limitations elaborated on.

(18) Ln 412: Here and throughout, I think it would better to refer to the validation process as to validating the whole system instead of the Viewer 2 software.

Answer:
The first paragraph of the discussion was adjusted to cover both parts of the JAM-R validation and assessment.

It now reads (lines 472-475): “By recording feeding and ruminating behaviour on pasture and in the barn, JAM-R was validated for sheep and goats. The classification performance of the Viewer2 software was assessed by behaviour observation. By using the system in a feeding experiment, issues during the application, such as technical failure rates and welfare issues, were assessed.”

Addition: Paragraphs 538-555 were adjusted to discuss the results of the new focus of the assessment of the application. The paragraph discussing the comparing values of feeding and rumination durations was shortened.

(19) Ln 512: Change reference header to English.

Answer:
done.

**Figures and tables:**

(20) Figure 2: My recommendation would be to edit this figure with new headers in English to avoid a lengthy figure legend with translations.

Answer:
Figure 2 is now presented in english.

(21) Figure 4: Although these findings have merit and are interesting in it’s own right, this figure does not contributed to answering any of the aims set out in the beginning the manuscript unless compared to manual observation. As it stands, the results presented are bound to be more linked to housing and feed rather than the technology.

Answer:
The focus of the application experiment was changed in relation to further issues of applicability. However from our point of view, the issue of plausibility of the output data provided by the Viewer2 (summary of feeding behavior per day and distribution over day) is part of that question. We therefore would argue for keeping Figure 4.

(22) Table 5: I don’t know if this particular table is of value in the light of the scope of the manuscript unless the difference between the technology estimation and true consumption is presented. The difference in consumption between species seem like a stand-alone result outside of the current aims. As it stands, the results presented are bound to be more linked to housing and feed rather than the technology.

Answer:
Some of its information was transferred into the text (lines 432-434) to allow for evaluating the plausibility and comparison of its values with the literature in the discussion. The table is deleted. A new table 5 now presents the variance of random effects, including the data logger, of the linear mixed effects models of three Viewer2 output variables.

# Answer to Reviewer #2

This study is interesting and valuable for investigation of animal behavior and other fields. However, it is not suitable for acceptation in the present form because of the important issues followed:

Answer:
Thank you very much for your valuable feedback and recommending our work for publication. We have revised the manuscript, taking careful account of all your comments.

(1) The authors did not clearly describe the procedure for this investigation sequently in Method, especiallly for the acheivement and processing of the raw data. If possible, why not present a overview of the data processing? In addition, I am also not clear how the Viewer2 work in the present study in this version of manuscript. Is it just a software for data statistic analysis? I think the authors should make some explanation about the Viewer2 in this respect.

Answer:
The Material and Methods and the results section were reorganized and some information added (See new table of contents below). More details on how the Viewer2 software works are provided in section 2.3.2 lines 232-248. We hope to have clarified now the processing of the raw data.

**2	Material and Methods	7**

2.1	Animals and housing	8

2.1.1	Non-experimental condition	8

2.1.2	Pasture	9

2.1.3	Experimental pens	10

2.1.4	Feeding experiment for application of JAM-R	11

2.2	Behaviour recording by observation	11

2.3	Automatic behaviour recording	13

2.3.1	MSR-jaw-movement recording system (JAM-R)	13

2.3.2	Viewer2 behaviour classification software	15

2.4	Comparison of the behaviour recording methods	16

2.4.1	Data management	16

2.4.2	Parameters of agreement	17

2.4.3	Effects of species, condition and recording frequency on agreement parameters	18

2.5	Application of JAM-R in a feeding experiment	20

2.5.1	Description of the feeding experiment	20

2.5.2	Assessment of welfare issues and data quality	21

2.5.3	Estimation of variance of data loggers	22

**3	Results	22**

3.1	Comparison of the behaviour recording methods	22

3.2	Parameters of agreement	24

3.2.1	Effect of condition: Pasture versus barn (10 Hz)	25

3.2.2	Effect of recording frequency: 10 Hz versus 20 Hz	25

3.3	Application of JAM-R in a feeding experiment	26

3.3.1	Values of feeding and ruminating behaviour	26

3.3.2	Assessment of welfare issues and data quality	26

3.3.3	Variance of data loggers on output variables of the Viewer2	27

(2) The aim of this study seems to validate the performance of the Viewer2 software in classifying the feeding and ruminating behavior in sheep and goat, using the observation by video as the standard for calculation of the efficiency in the system of JAM-R and Viewer2. However, I could not find comparison between the two methods in context. In addition, the data from video observation should also be displayed in supplementary files.

Answer:
We compared each second of the Viewer2 behaviour classification to the same second of the human behaviour observation classification for (non-)accordance (Lines 264-267, Table 2). From this data the four parameters of agreement were calculated (Section 2.4.2 Lines 287-304) that were further analysed (Sections 2.4.3, Lines 305-335).

For better clarification we reorganized Material and Methods and renamed some headers (see table of contents above). To sum it up, we summarized all information of the behaviour observation in section 2.2 (lines 178ff), all the information on the automatic behvaiour recording and classification system in section 2.3 (lines 205ff) and then described the comparison of these methods in section 2.4., which now reads “Comparison of the behaviour recording methods” (lines 254ff). Additionally, we included the file of comparison of each second of observation to the supplementary data.

(3) Further more, there were some errors in grammar, i.e. line 257-258, as well as formats of typings in the manuscript.

Answer:
The manuscript was professionally proof-read. The revised version was again professionally proof-read.

# Answer to Reviewer #3

This review is for the manuscript titled “Automatic monitoring of feeding and ruminating behaviour of sheep and goats”.

Answer: Thank you very much for your valuable feedback and recommending our work for publication. We have revised the manuscript, taking careful account of all your comments.
 **Major comments**

1) Naturally, goats are browsers and sheep are grazers where their natural grazing behavior can be assessed. Can the authors provide the reason why they used goats on pasture grazing to evaluate feeding behavior study which may not reflect the natural behavior of the animal, especially under grazing condition?

Answer:

In his classification of ruminant species, Hofmann describes goats as intermediate species, which browse and graze (Hofmann, 1989^[1]^). Grazing is therefore part of their natural feeding behavior. The pasture used for the validation was a natural meadow (see lines 134-136), that allowed browsing to some extent. Under farming conditions, dairy goats are commonly fed on pasture or in the barn. As we did not see relevant differences in the parameters of agreement between species (lines 396-400) we would argue that we were able to assess the feeding behavior of both species equally good for these two conditions. If the system also works for goats under extensive conditions would still have to be shown.

We now mention this restriction in the discussion (Line 507-511): “To cover the full potential of the JAM-R, further aspects need to be investigated. For example, the classification performance in recording periods, where feeding in the barn and on pasture must be distinguished, or the applicability in natural environments, where the predominant feeding activity is browsing rather than grazing.”

2) For the JAM-R recording in feeding pens, a one day recording for 10 and 20 Hz recoding was analyzed (L198-99). Is there any pervious analysis that may suggest the one day recording can generate sufficient data for analyzing the feeding and ruminating behavior in pen situation?

Answer:
To our knowledge there is no previous analysis available in the literature that would answer how many seconds of recording would be necessary. The observation period in the barn was carefully chosen between 11:00 to 14:00, as this was right after feeding (11:00 am) and was known to include the rumination phase around noon. The sheep and goats were observed to feed on average for 2107 seconds (standard deviation ±1068 s) and ruminate on average for 2678 (±1805) s during these three hours of the observation days in the barn. Furthermore, five animals per species were used to account for variation that might result from individual behavior in feeding. Based on these seconds the parameters accuracy, specificity, sensitivity and precision and their confidence intervals were calculated. As the estimates of the confidence intervals are rather small, (Fig. 3) we would argue that the data set used was at least sufficient for the conditions investigated. For the effect of frequency on the parameters of agreement, 38 observations were used to estimate the effect of two main effects and their interaction in the linear mixed effects model, which leaves sufficient degrees of freedom for the calculation of these effects.

We included the samples size of the data set and subsets in the model formula (lines 313, 322, 329).

We included this topic in the discussion (Line 479-482): “[…] 10 Hz already provides a good estimate of these behaviours for dairy sheep and goats under the conditions investigated. However, an increase in recording frequency from 10 Hz to 20 Hz slightly improved the classification performance of the Viewer2 in this study.”
And (line 487-489): “The effect of the recording frequency on the parameters of agreement needs to be tested under additional conditions, such as pasture.”

3) While analyzing the application data, at what recording frequency was the data summarized (min, sec, hr? Line 393) and was that treated as a repeated measure in the model for analyses?

Answer:
Recording frequency was generally 10 Hz (= 10 recordings per second), except for a few recordings in 20 Hz (Lines 342-344). Every single recording is then classified as “feeding”, ‘ruminating’ or ‘other’ by the Viewer 2. The durations for feeding and ruminating per day are calculated by summarizing these classified recordings. The sums are expressed in minutes per recording period (lines 238-239). In case of the feeding experiment the recording period was 24 h (lines 344-345) so the data is expressed as min/d and was calculated to hours/day for a better overview.

The model in this section was changed to report the variance of the random effects. The included random effects are the data logger, the individual, the feed, the date and the species. The random effect of frequency was not included in this model. This variation is covered in the random effect of data loggers, as only two specific of the 16 data loggers used, were able to record on 20 Hz. For Figure 4 the data was split per hour to visualize the mean feeding and ruminating durations per hour over the day.

To make this clearer we rephrased the paragraph in M&M.
It now reads (lines 342-345): “The feeding and ruminating behaviour of each animal was recorded with the JAM-R system on experimental days 4, 5, 9 and 10. The recording frequency was set to 10 Hz, except for 47 cases in which it was set to 20 Hz for the validation described in Section 2.4, which were recorded during the last MR of this experiment. The recording period was set to 24 h from 14:00 to 14:00 the next day”.

And (line 372-377): “In the next step, the order of magnitude of the variance attributed to the individual data loggers (n = 18) was estimated. Three output variables of Viewer2, the feeding duration in hours per day, rumination duration in hours per day and mastications per cud (MpC), were analysed each in a linear mixed effects model (lme4 package, REF) to estimate the variance of the random effect data logger. Other random effects for comparison included in the model were individual, species, the feed (HH, GH, MG) and the date.”

**Minor comments**

**Abstract**

1) Line 39-41: review as “… system with the Viewer2 software provided a reliable technology for automatic recording of feeding and ruminating behavior of sheep and goats on pasture and in the barn”.

Answer:
done.

**Introduction**

2) The introduction section is a bit long and requires revision to make it concise and also increase its flow.

Answer:
We shortened the section related to the use of behaviour parameters (Lines 44-51).

3) Line 45: revise as “… monitoring feeding and ruminating behavior of ruminants is …”

Answer:
Changed to (lines 43-44): “In ruminants, monitoring feeding behaviours is useful for assessing their health and welfare.”

4) R3: Lines 61-64: please revise this sentence is not clear

Answer:
As this section of the introduction was shortened, this sentence was removed.

5) R3: L65; … feeding and rumination ….

Answer:
To keep the wording concise, we use “ruminating” throughout the entire manuscript.

6) R3: L69-73: this seems to be a single sentence and can be revised as “…(15) including; 1) …; 2)…..; and 3)…”

Answer:
We decided to to avoid sentences that span over more than three lines. We rephrased the section. It now reads (lines 57-63): “Devices that record actual jaw movements with pressure-sensitive sensors have been designed repeatedly because this approach has a number of advantages (9). First, it measures behaviour directly at the individual level, so the data can always be linked to the individual, independent of the animal’s location (barn or pasture). Second, the available sensors are small enough to fit into common head collars, so animals can easily adapt to the system. Third, the measurement of jaw movements is at the exact location where the behaviour of interest originates.”

7) L74-76: revise as “… feeding and ruminating behaviours as the duration and frequency of mastication differs.”$

Answer:
We rephrased the sentence. In now reads (lines 64-66): “This enables a distinction between feeding and ruminating, as the duration and frequency of mastication differ between these two behaviours.”

8) L79: revise as “… (18). The system was tested and validated for dairy cows (19).”

Answer:
We rephrased the sentence. It now reads (lines 69-70): “The JAM-R technology has previously been tested and validated for dairy cows.”

**Material & Methods**

9) L108: revise “To validate the software Viewer2, feeding and ….”

Answer:
We rephrased the sentence. It now reads (lines 98-100): “To validate the JAM-R, the behaviour classifications of Viewer2 software (described in Section 2.3.2) were compared to the gold standard method of behaviour observation (described in Section 2.2).”

10) L116: delete respectively

Answer:
The paragraph was revised. It now reads (Lines 102-105): “The agreement between the classifications of Viewer2 and the behaviour observations was evaluated. Additionally, JAM-R was applied in a feeding experiment with multiple 24 h recordings and assessed for technical and welfare issues.”

11) L121-22: revise this sentences. Is this means 2 goats (from pasture) for observation, 5 goats (including the two) for video, and 5 sheep for video monitoring used? Also, clarify if the animals used for monitoring behavior on pasture and pen settings were the same.

Answer:
We rephrased the sentence. It now reads (lines 151-154): “Video recordings of five sheep and five goats ([…]) were collected [...]. The animals were not the same as those used on pasture, apart from two goats that were used in both situations.”

12) L114-131: include the average body weight (±SD) of the experimental sheep and goats

Answer:
The information was added (lines 116-117): “The mean weight of goats was 67.7 (SD ± 7.5) kg and of sheep was 79.1 (± 7.7).”

13) L140: revise “… pend had three drinking water sources and one mineral supply site.”

Answer:
done.

14) L156: Is there a reason why the sheep were moved to the same plots that is already used by the goats earlier?

Answer:
This was necessary to enable water supply. However, sheep received additionally fresh pasture for habituation and during the experiment. (Line 147)

15) L164-65: just out of curiosity, what is the value of having a round table in the pens?

Answer:
The elevated tables and platforms are provided for goats to structure the pens to give possibility for the goats to avoid each other and give climbing possibilities. For further information see Aschwanden et al. 2009a^[2]^ and Aschwanden et al. 2009b^[3]^.

16) L165: revise as “ Experimental animals received a MR containing 55% …… ad libitum.”

Answer:
Done.

17) L167: Naturally, goats are browsers and sheep are grazers where their natural grazing behavior can be assessed. Can the authors provide the reason why they used goats on pasture grazing to evaluate feeding behavior study which may not reflect the actual natural behavior of the animal?

Answer:
See Answer to the Major Comment 1).

a) L172-73: revise as “… on the weather condition, ….”.

Answer:
Done.

b) During higher temperature, animals tend to be less active and gather under a shade/ hut and this is one of the occasions that rumination takes place. So, during grazing in the field, is the observation in the morning specifically focus on feeding and the one in the afternoon focused on rumination?

Answer:
The animals grazed and ruminated in several phases throughout the whole day. We observed in observation periods of 1.5 to 3.5 hours. Most of the time both behaviors could be observed in such an observation period.

19) L177: which previous experiment? Include the source. This may also answer the question if the two days of recording can generate sufficient data for behavioral study. As indicated in L198, basically it is a one day data as different recording frequency (10 vs 20 Hz) was used on separate days.

Answer:
The data for validation in the barn was collected during testing the third mixed ration in the feeding experiment described in section “2.5 Assessment of application of JAM-R in a feeding experiment”. During enrolling the first two mixed rations we assessed during which hours the main phases of feeding and rumination will occur. From this information, we chose the period 11:00 to 14:00.

To make this clear, we added the information.
Lines 151-153: “Video recordings of five sheep and five goats […] were collected during the feeding experiment which will be further explained in Section 2.1.4.”
Lines 176-177: “During the experimental phase of (the last MR) MG, videos for behaviour observations (Section 2.2) were recorded.”

20) L205: different font

Answer:
Corrected.

21) L218: revise as “ …file (one per animal per day) ..”

Answer:
It now reads (lines 229): “**the single MSR files** must be uploaded to Viewer2.”

22) L226: Based on L144 L160, and L301, all the grazing and pen feeding experiments were conducted in 2020 however, in the excel spreadsheet provided here (under application tub in the file) the date stamp indicated data collected in 2019.

Answer:
The experimental time was incorrectly stated. The experiment for the application was conducted from October 2019 to April 2020. This was corrected. (Line 165)

23) L300: Is this a separate experiment from the one stated in L159-66 for pen feeding that used just five animals, March to April 2020?

Answer:
This was actually the same experiment. We clarified this. Please see answer to point (19).

24) L306-7: Explain the reason for providing different MRs? Also, revise – a 14 days adaptation period was mentioned on L314 – so perhaps a specific MR was provided for a total of 24 days (14 adaptation and 10 experimental).

Answer:
The objective of the feeding experiment required that three different MRs common in small ruminants under intensive housing conditions were studied. It additionally allowed us to collect data for the validation of the JAM-R-system during feeding of the third MR.

The sections of M&M were restructured according to the comments of Reviewer 1. We hope to have clarified these issues now.

Information on the feeding experiment is now in Section 2.5. Every ration was fed for 10 days with a preceding habituation phase of 14 days. (Lines 174-176)

25) L324-5: what was the recording frequency considered for JAM-R (min, sec, hr? L393) and was that tread as a repeated measure in the model for analyses?

Answer:
See answer to Major comment 3)

26) L326: please include the quality analysis results for the grazed pasture as well

Answer:
According to the modifications of this section suggested by Reviewer 1, information on the quality analysis of the MRs was moved to the supplementary section. There are no specific analysis for the pasture available. Pasture quality is described in lines 134-136. For the validation of the system, we investigated if the parameters of agreement differed between pasture and the barn (which was based only on the third MR) (Line 162). Potential effects of feed composition on the parameters of agreement would have to be tested in a follow-up experiment based on specific hypotheses. This was not the scope of our study. Concerning the results of the application in the feeding experiment, an influence of the different qualities of the MRs on the outcome variables (feeding duration, rumination duration and bites per cud) can be expected and the effect of feed was included as random effect. (Lines 380, Results Tab. 5).
No changes made.

Results

27) L341: The system seems a poor predictor for drinking (Table 4) especially on pasture. Include descriptions on drinking behavior as well.

Answer:
The system does not predict drinking behaviour. According to the specific pressure patterns “feeding” and “ruminating” is classified. All seconds non-classified as feeding or ruminating are considered as “no / other behaviour”. The correct classification for drinking behaviour would be “no/ other behaviour”. In our observation we included drinking to see whether it would be correctly classified as “no/ other behvaiour”.

The explanation of the Viewer2 software and its classification were reformulated and expanded (see Section 2.3.2). We also rephrased the information in the results section (lines 387-389): “The seconds at which no behaviour or other behaviour was observed and the software should “ignore” were correctly classified as “no or other behaviour” in 89.5% of seconds (Table 4).”

28) L347: revise as “… were not correctly classified …”. As the system depends on change in pressure (with no visual aid to specifically identify and label activities), the observed

Answer:
See answer to comment 27.

29) L373 & 384: include the details of the results reported under these sections in a table form, perhaps expanded on Table 4 or added as supplementary table.

Answer:
The statistical results were added as Supplementary Table 3.

30) L390: revise as “On average (±SD), sheep fed for 5.1….. MpC across the three MRs. Conversely, goats fed ….”

Answer:
Done.

31) L403-4: On L303-4, stated that data was collected from 26 sheep and goats but only results from 24 is reported here.

Answer:
There are 24 sheep and 24 goats as experimental unit of the experiment, but two per species were replaced during the experiment.

The information was added in the methods section (lines 168-169).

32) L512: References

Answer:
Done.

**Tables**

33) Table 5: Include the unit for dry matter intake

Answer:
This table was excluded in the new version of the manuscript. The DM intake information with its unit was included in the text.

It now reads (lines 432-434): “On average (± SD), sheep consumed 1.34 (± 0.13) kg DM in 4.9 (± 1.0) h/day, ruminated for 7.2 (± 1.4) h/day and had 70.43 (± 10.7) MpC across the three MRs.”

34) Supplementary 1 (word file)
    The reported size of area and the numbers placed on the demarcated landscape doesn`t match. For example, for observation goats site the number on the map is 100 m by 119.55 m but the reported area 503.58 m2. The same for the sheep sites as well.

Answer:
The circumference of the plot “observation goats” is 119.55 m as it is stated in the box below and the 100 m point was marked. We removed this mark for 100 m to avoid this confusion.

35) Supplementary (excel file)
    As stated above, the data collection year needs to be checked (2019 vs 2020). The headings for individual columns within each tabs needs to be clearly presented/stated. For example, in the ‘application’ tab ‘starttime’ in column A is indicated as 140000 and in column J and K separate date and time reported.

Answer:
The experimental phase of the application was stated wrong. It is corrected and lasted from October 2019 to April 2020 (line 165). The supplementary data table was reorganized: The column ‘starttime’ was deleted. It was from the original file, which is not used in the analysis.

1. Hofmann, R. R. (1989). Evolutionary steps of ecophysiological adaptation and diversification of ruminants: a comparative view of their digestive system [journal article]. *Oecologia*, *78*(4), 443-457. <https://doi.org/10.1007/bf00378733>
2. Aschwanden, J., Gygax, L., Wechsler, B. and **Keil**, N.M., (2009) Loose housing of small goat groups: Influence of visual cover and elevated levels on feeding, resting and agonistic behaviour. Appl. Anim. Behav. Sci. **119**, 171-179.
3. Aschwanden, J., Gygax, L., Wechsler, B. and **Keil**, N.M., (2009) Structural modifications at the feeding place: Effects of partitions and platforms on feeding and social behaviour of goats. Appl. Anim. Behav. Sci. **119**, 180-192.
