# Supplementary material for: Validation of automatic monitoring of feeding behaviours in sheep and goats
Source: PLoS One. 2023 May 18;18(5):e0285933. doi: 10.1371/journal.pone.0285933 (PMC10194855; doi:10.1371/journal.pone.0285933)
Supplement: Attachment — Submitted filename: Answer to the reviewer_2.docx [file pone.0285933.s006.docx]

**Round 2. Reviewer 1**

Reviewer #1: General comments: First off I would like commend the authors for the great effort of revising this manuscript. I find that the new structure looks better and the manuscript has improved with the new additions and clarifications.
That said, I do still have some additional concerns with the manuscript in its current state, in particular the data cleaning section which could have implication on both the current results and its discussion. Thank you for the effort so far.

Please see line by line below.

Answer:

*Thank you very much for your valuable feedback and recommending our work for publication. We have revised the manuscript, taking careful account of your comments.*

1) **Line 55-57. I would consider writing out examples of these mechanisms.**

Answer:
We added examples (line 58 – 60): “In the past, automatic monitoring systems were developed based on a variety of mechanisms, like head position with accelerometers or jaw movements with acoustic or pressure sensors.”

2) **Line 98-100. I would consider clarifying briefly what the gold standard is in this paragraph. I would also make sure the reader understands what the gold standard is in section 2.2. Currently you describe both manual live observation and manual observation from continuous recordings, but you don’t define the gold standard. Please clarify.**

Answer:
We added the definition of the “gold standard” in the introduction (lines 53 – 58): “Despite the rise of technological solutions, behaviour observation by human observers is still commonly used to record animal behaviour and can be considered the “gold standard”. However, it requires training and is very time consuming, making it challenging in research and very demanding on farms.

Automated recording methods can substitute the human observer provided they are equally good at detecting feeding behaviour as humans.”

We rephrased (line 102 – 104): “To validate the JAM-R, the behaviour classifications of Viewer2 software (described in Section 2.3.2) were compared to the gold standard of behaviour recording, which is the observation by a human (detailed methods used is described in Section 2.2).”

We added in section 2.2 (lines 185 – 187): “During the behaviour observations which is considered the gold standard, all animals were equipped with the reference behaviour recording method - the JAM-R.”

3) **Line 152-153. Why did two goats re-enroll in the project? Were they simply filler animals to keep the group size intact?**

Answer:
*There is no particular reason why the two goats were re-enrolled in the project; it was a coincidence that they were included in both situations*. *We had videos from most of the animals. Unfortunately, many videos had a bad quality in terms of lighting or because the head of the animal was hidden behind the other animal or barn structures for some time. The animals chosen were simply those, where we had videos of two required days (once 10 Hz and once 20 Hz) that could be coded (= good lighting and animals head in sight the entire time).*

We rephrased the information of the animals chosen for the barn validation (lines 154 – 159): “During the feeding experiment, which will be further explained in Section 2.1.4., videos were recorded. Videos of five sheep and five goats (LA = 4, EF = 1, CC = 2, SA = 3) were chosen based on the availability of sufficient video image quality (e.g. lighting, animal in sight) for continuous behaviour observation. This included two goats that were also used in the pasture situation and three other goats and five additional sheep.”

4) **Line 179-180. I think it would be more clear if you simply wrote out 6h instead of 21,600 seconds. Were the videos 3h each on the same time of day across two days, or on two occasions on the same day? How were these periods selected, by choosing parts of the day when the sheep and goats where most likely to display the behaviors in the ethogram? Was it during the same time frame as written later on lines 190-193?**

Answer:
*We indeed checked each single of 21’600 seconds. They were coded from videos of two days of 3 hours of two different animals. We moved this section below the explanation of the video observation procedure, where it is explained which periods were chosen and why and rephrased for clarification.*

It now reads (lines 206 – 210): “The behaviour observations on pasture (direct) and in the barn (by video) were conducted by one trained observer (A.D.). Two of the videos were coded again by another trained observer (R.B.) and each second (21’600 s) was assessed for agreement. R.B. reached an agreement of 97.6–98.2% for seconds observed as feeding by A.D. and 98.5–99.9% for seconds of rumination of A.D.”

5) **Line 196: Is 2h of non-continuous data per animal enough to properly validate the logger?**

Answer:
*The data was recorded continuously. Every 10 min we switched to another animal adding up to 22.8 hours (sheep) and 22.7 hours (goat) of continuous observation in total. The validation of the software Viewer2 is therefore based on the classified seconds of 45,5 hours (22.8 + 22.7 h) of continuous recording (see point 6). In Section 2.5, we focus on the variance between the loggers, which is based on 401 24h recordings during the feeding experiment, summing up to 9’624 hours.*

No changes made.

6) **Line 189-203. If I understand correctly the animals on pasture were not recorded using a gold standard, as a 10 minute intervals were used, compared to the pens which had continuous recordings from video? If continuous interval focal animal sampling is the gold standard for goats and sheep on pasture, please be very clear in the manuscript and provide a reference.**

Answer:
*Apparently we were not clear enough. We rephrased that sentence and included, that the 10 min intervals of pasture observation were indeed also continuous 10 min behaviour observations. Per animal data of 3 hours of video observation and 2.3 hours of direct observation were collected (Line 203 and Line 109).*

It now reads (line 195-197): “. Using focal animal sampling, the observer continuously observed the animals by switching between animals in 10 min intervals or when the head of the focal animal moved out of sight.”

7) **Line 218: “The data loggers HAD to be programmed…”**

Answer: *done.*

8) **Line 218-221: Did you ever consider looking at higher frequencies as well? You mentioned earlier that the data logger could be programmed up to 50 hz.**

Answer:
*As we only had access to an older generation of MSR loggers for this project, our loggers were not able to record on 50 Hz. As discussed in lines 498 – 500, we suggest the comparison of higher frequencies for future research (“For a more detailed analysis of ruminating and feeding behaviour, and depending on the research question, a recording frequency of 20 Hz (or even higher) could therefore be advisable.”)*

No changes made.

9) **Line 221-223. While re-reading the manuscript, I got curious to why the activity was omitted from the manuscript?**

Answer:
*This data was not omitted, we did not collect it. Measuring activity was not within the scope of this study and we needed the available memory capacity for the 20 hz recordings.*

No changes made.

10) **Line 223-225. So this is quite the limitation of this particular logger if you wanted to record continuous data over a longer period of time without disturbing the animals. Would there be enough utility for this logger when you factor in the task of changing these loggers daily? In line 226, you mention other loggers, do you mean from the same company/brand with longer battery life, or other loggers from other companies? If the latter, you might want to be careful in how this limitation is portrayed as you are essentially promoting other companies loggers with longer battery life. Especially since the company co-authoring the manuscript.**

Answer:
*For sustainability reasons, we use electronic equipment as long as it is functioning. Therefore, the loggers used in this experiment were comparably old (purchased 2009 - 2010, see line 449). Newer loggers are available from MSR electronics GmbH. This information was included.*

It now reads (lines 230 – 234): “The data loggers (in the version available for this project) had to be removed from the animals to save the recorded data (as an MSR file) and reprogrammed for the next recording period once per day due to the data storage capacities of the loggers being a maximum of 29 hours. However, newer versions of the data loggers are available with larger data storage capacities for longer continuous recording periods from MSR electronics GmbH.”

11) **Line 255-258. So if I understand correctly, the data from the pasture on 10 hz was compared to a gold standard, and for animals in the pen, both a 10 hz and a 20 hz data set was compared to a gold standard.**

Answers:
*This is correct. The automatically recorded data classified into behavior by the Viewer2 was compared to the behaviour recorded by the human observer.*

We added an introductory sentence in this section (lines2– 267): “The behaviour recorded by the human observer (directly on pasture or from video in the barn, see Ethogramm in Tab. 1) was compared to the behaviour recorded at the same time with the data loggers and classified by the Viewer2. The data loggers were set to a recording frequency of 10 Hz when the animals were on pasture. In the barn, the recording frequency was alternated between 10 and 20 Hz, so every animal had one day of recording at 10 Hz and one day of recording at 20 Hz.”

12) **Line 264-266. Please be clear with defining this ‘gold standard’ is.**

Answer:
We rephrased (lines 275 – 276): “This resulted in numbers of correctly and incorrectly classified seconds, taking the behaviour observed by the human as the true value (“gold standard”).”

Please also see our answer to point 2).

13) **Line 306-310. Consider breaking this sentence up for clarity. Also consider replacing the word “checked” for ‘controlled’ depending on the new sentences.**

Answer:
We changed to (lines 315-318): “With the complete dataset, in linear mixed effects models (*lmer* of lme4 package, (24); one per parameter), as outcome variables, each parameter of agreement was estimated in dependence of the fixed effects species (sheep and goat) and behaviour (feeding and ruminating). The models also considered the repeated measurements of the individual, the effect of the condition and the recording frequency by random effects.”

14) **Line 363-364: By excluding these errors, are you not excluding data that would tell you the performance of these loggers, i.e. selection bias?**

Answer:
*As reported in the first part of the paper (the validation of the Viewer2), the software does not classify behaviour 100 % correctly. Therefore any dataset generated with the JAM-R, needs to be checked for implausible values to improve data quality.
In this section of the paper (application in a feeding experiment), our purpose was to provide the reader (that potentially wants to use the system to record feeding behaviour) with information how much data loss, technical failure and variation in the data caused by a set of these data loggers can be expected; or better: need to be considered. From our point of view to report on the variation in the data caused by the loggers, the information is more useful after identifying and removing implausible values. The procedure must of course be explained and the number of removed data has to be documented (line xx-xx).*

We added information to explain better the aim of the application in a feeding experiment (lines 346-350): “To assess issues of the applicability of the JAM-R, it was tested in a feeding experiment. Issues for animal welfare due to wearing the halters were recorded. Data loss events were reported which were caused by technical failure, animal welfare issues or human error. Raw data of the feeding and rumination durations were inspected for plausibility. The variation in the data that was caused by this set of data loggers was assessed.”

1) **Line 364-370: You state that some of the feeding and ruminating durations of less than 3.1h/d were implausible and that the cut-off values are based on minimal durations for feeding in goats and small ruminants. Under what conditions? The same? The Lu reference refers to an indoor feeding trial, and Penning recorded pasture based feeding durations of up to 11.4h for goats and 12.6h for sheep. Way over the exclusion threshold made by your outlier calculation. Then you state that implausible high durations for feeding and ruminating were identified by outliers, and in the next sentence you state that no outliers were found. This would be the case if ‘implausible values’ were omitted from the data set as previously stated.**
2) **You might want to revise/control how this data set was cleaned and using what rationale. Ergo, perhaps the data omitted was not implausible after all? I would consider looking at this data again to make sure the calculated accuracy, specificity and precision are valid and derived from a correctly cleaned data set.**

Answer:

1) *We aimed at choosing cut- off values for implausible values as conservatively as possible and specifically tailored to our experimental approach. For implausible low values, we took the minimal duration that we could find for small in the literature. Reports on maximum durations are rare, in a broad range and do not match our experimental conditions (indoor vs pasture, individual vs group housing, different feeds). For this reason, we used the mathematical definition of outliers for implausible high values. For rumination, we did not have such outliers in the data.*
2) *For the calculations for accuracy, sensitivity, precision and specificity we compared the classifications of single seconds and did not calculate durations. Therefore, we had no implausible values and did not exclude any data in that section of the paper.*

We clarified that the used cut-off values are not a general approach, but based on our experimental approach. It now reads (lines 373-382): “Thereafter, the Viewer2 output data files were analysed for implausible values that would compromise data quality. It was assumed that data files of extreme values for feeding and/or ruminating duration would indicate incorrect classifications in these specific files. For the purpose of the experimental approach of this feeding experiment, data files with feeding and ruminating durations of less than 3.1 h/d were deemed implausible. These cut-off values were set from the minimal durations for feeding in goats reported by Lu (26) and for ruminating in small ruminants reported by Penning et al. (27). Due to the lack of suitable literature, implausible high durations for feeding and ruminating were identified by outliers (+/-1.58 interquartile range/square root (n), (28)), which excluded feeding durations exceeding 8.1 h/d and rumination durations higher than 10.7 h/d.”

For clarification, we now state all number of outliers for feeding and rumination duration in the results section (line 475-478). “From the remaining 510 MSR files that were analysed by Viewer2, 109 (21.4%) output data files were identified to have implausible high (feeding: 23; ruminating: 0) or low (feeding: 25; ruminating: 81) durations of feeding and ruminating, resulting in a final dataset of 401 data files. This represents 71.4% of the executed 562 MSR files.”

16) **Line 369. Square root.**

Answer: *done.*

17) **Line 442-461. Not**

Answer:
*We assume you intended to write more. Please clarify.*

18) **Line 458-461. See previous comment for lines 364-370. I think the majority of this paragraph can be condensed and merged in to section 2.5.2 and the few welfare aspects regarding the logger placement can be discussed in the discussion.**

Answer:
*We would argue that these results are part of the technical evaluation next to the logger variation and prefer to keep it here. See also our answer to comment 14).
We rephrased parts of the associated methods section and this results section and the respective headings (2.5.2 and 3.3.2) to be clearer about the purpose of these sections.*

The section headings were change to (line 361 + 454): “Assessment of welfare issues, failure rate and data plausibility”

It now reads (lines 369– 382): “It was noted daily, if a recording was not executed due to human error or animal welfare issues. Furthermore, all technical failures during the feeding experiment from all three MRs were recorded. These were identified by the number of MSR files that were not readable by Viewer2.
Thereafter, the Viewer2 output data files were analysed for plausibility that would compromise data quality. It was assumed that data files of extreme values for feeding and/or ruminating duration would indicate incorrect classifications in these specific files. For the purpose of the experimental approach of this feeding experiment, data files with feeding and ruminating durations of less than 3.1 h/d were deemed implausible. These cut-off values were set from the minimal durations (known to the authors) for feeding in goats reported by Lu (26) and for ruminating in small ruminants reported by Penning et al. (27). Due to the lack of suitable literature, implausible high durations for feeding and ruminating were identified by outliers (+/-1.58 interquartile range/square root (n), (28)), which excluded feeding and ruminating durations exceeding 8.1 h/d and 10.7 h/d, respectively.”

This paragraph was moved from Materials and Methods to the results section (now lines 465-469): “From the 576 scheduled MSR files, in 14 cases (2.4%), the MSR file was missing due to human error (data loggers were unintentionally not programmed) or animal health (one animal could not wear the halter for the entire experiment due to an abscess on the jaw, which ruptured when trying on the halter the first time and then needed to heal), resulting in the analysis of 562 24-hour MSR files.”

19) **Table 2. Make sure to clarify what type of video recording that was used. Continuous?**

Answer:
*We clarified that both conditions of behaviour observation were continuous observations (see point 6) and added this information in the description of table 2 (line 294).*
